# Early cellular signaling responses to axonal injury

**DOI:** 10.1186/1478-811X-7-5

**Published:** 2009-03-13

**Authors:** Thomas J Lukas, Ai Ling Wang, Ming Yuan, Arthur H Neufeld

**Affiliations:** 1 Forsythe Laboratory for the Investigation of the Aging Retina, Northwestern University Feinberg School of Medicine, Chicago, IL 60611, USA; 2 Department of Ophthalmology, Northwestern University Feinberg School of Medicine, Chicago, IL 60611, USA; 3 Department of Molecular Pharmacology & Biological Chemistry, Northwestern University Feinberg School of Medicine, Chicago, IL 60611, USA

## Abstract

**Background:**

We have used optic nerve injury as a model to study early signaling events in neuronal tissue following axonal injury. Optic nerve injury results in the selective death of retinal ganglion cells (RGCs). The time course of cell death takes place over a period of days with the earliest detection of RGC death at about 48 hr post injury. We hypothesized that in the period immediately following axonal injury, there are changes in the soma that signal surrounding glia and neurons and that start programmed cell death. In the current study, we investigated early changes in cellular signaling and gene expression that occur within the first 6 hrs post optic nerve injury.

**Results:**

We found evidence of cell to cell signaling within 30 min of axonal injury. We detected differences in phosphoproteins and gene expression within the 6 hrs time period. Activation of TNFα and glutamate receptors, two pathways that can initiate cell death, begins in RGCs within 6 hrs following axonal injury. Differential gene expression at 6 hrs post injury included genes involved in cytokine, neurotrophic factor signaling (Socs3) and apoptosis (Bax).

**Conclusion:**

We interpret our studies to indicate that both neurons and glia in the retina have been signaled within 30 min after optic nerve injury. The signals are probably initiated by the RGC soma. In addition, signals activating cellular death pathways occur within 6 hrs of injury, which likely lead to RGC degeneration.

## Introduction

Axonal damage of long projecting neurons leads to retrograde degeneration and loss of the soma of the neuron by programmed cell death over a period of several days. However, little is known about the timing of events in the soma of the neuron immediately following damage to the axon and how soon the affected neuron with axonal damage signals surrounding glia and other neurons of the catastrophic event.

Retinal ganglion cells (RGCs) are long projecting neurons whose axons make up the optic nerve. To determine the temporal sequence of cellular signals and interactions following axonal injury, we have used optic nerve crush. The somas of the RGCs are in a single layer in the retina and are easily sampled and visualized. Similarly, the glia and other retinal neurons that are associated with the RGCs are also in specific layers and can be readily observed by immunohistochemistry.

Most studies of optic nerve damage leading to RGC loss make observations of the changes in gene expression, the level of a particular molecule or activation of a pathway starting at 24–48 hrs following the injury [1-3]. For example, inactivation of phospho-AKT and phospho-BAD have been reported as early as 48 hrs after injury [1]. Upregulation of proapoptotic proteins such as BAX and BIM has been reported at 24 hr post injury [4-6]. Seven days after optic nerve crush, there is increased caspase-3 activity and significant loss of RGCs [6-8].

In the work presented here, we have looked for changes in the retina within 6 hrs following optic nerve crush. Our interest was not biased towards any one pathway that had changed, but to use any changes that we found to determine the timing of cellular events and cell to cell signaling, particularly those that may precede degeneration. Thus, the work presented here attempts to answer the following questions:

1. When does the soma of the RGC "sense" that its axon has been injured?

2. Which cells in the retina are signaled by the RGCs that a catastrophic event has occurred?

3. Are cell death signals apparent in the first 6 hrs following injury to the axon?

4. Are there nuclear events in the first 6 hrs following injury to the axon?

We used phosphoproteomics and microarrays to find early protein and gene expression changes following axonal damage that could be followed up with immunoblots, immunohistochemistry and RT-PCR. We then used our results to deduce a temporal sequence of cell to cell interactions and representative signaling events in the retina following optic nerve injury. We interpret our results to indicate that within 30 min of the axonal injury, the RGC soma has detected the axonal damage and has already signaled other cells throughout the retina. In addition, cell death pathways have been activated within 6 hrs of the axonal injury and changes in gene expression have been initiated by 6 hrs.

## Results

### Detection of phosphoproteins in the retina after optic nerve injury

We reasoned that phosphorylation of proteins would participate in initial responses; therefore, a proteomics approach was used to establish that there are phosphorylation events within the first 6 hrs following axonal injury. Database searching of the mass spectrometry data was used to identify the captured phosphoproteins (Additional file 1) and to obtain a differential, temporal spectrum of phosphoproteins. The detected phosphoproteins were further analyzed using Gene Ontology (GO) programs, GoMiner [9] and ProfCom [10] to identify "enrichment" of phosphoproteins within the context of categories of cellular pathways. Enrichment of phosphoproteins refers to detection of changes beyond expected from a random distribution. The "changed" p-values are based upon Fisher's test for significance [9]. Table 1 contains the GO categories of biological processes with respect to phosphoproteins that were significantly (p < 0.05) enriched, compared to control tissue, after axonal injury. Note that enrichment of phosphoproteins occurred in intracellular signaling, protein kinase, modulation of transcription and ion channel categories, among others.

**Table 1:** Gene Ontology Analysis of Phosphoproteins after Axonal Injury

GO ID	Total	Under	Over	Change	p-Value	Term
166	2142	7	20	27	0.0001	nucleotide binding
17076	1853	7	15	22	0.0014	purine nucleotide binding
30554	1533	6	13	19	0.0019	adenyl nucleotide binding
5524	1446	6	12	18	0.0024	ATP binding
7242	1200	5	8	13	0.0278	intracellular signaling cascade
16301	951	4	8	12	0.0118	kinase activity
5198	807	2	9	11	0.0092	structural molecule activity
16773	770	2	8	10	0.0175	phosphotransferase activity, OH acceptor
4672	669	2	7	9	0.0194	protein kinase activity
6468	673	2	6	8	0.0498	protein amino acid phosphorylation
30001	406	2	5	7	0.0113	metal ion transport
15672	321	1	5	6	0.013	monovalent inorganic cation transport
5216	353	1	5	6	0.0197	ion channel activity
15268	386	1	5	6	0.029	alpha-type channel activity
15267	407	1	5	6	0.0361	channel or pore class transporter activity
9966	443	2	4	6	0.0508	regulation of signal transduction
6813	168	1	4	5	0.0035	potassium ion transport
7017	216	3	2	5	0.0098	microtubule-based process
5261	254	1	4	5	0.0186	cation channel activity
3779	259	1	4	5	0.02	actin binding
5516	111	1	3	4	0.0045	calmodulin binding
5267	140	0	4	4	0.0101	potassium channel activity
3712	160	2	2	4	0.0158	transcription cofactor activity
5244	175	1	3	4	0.0212	voltage-gated ion channel activity

Table 2 presents a selected list of phosphoproteins that we culled from the enriched GO categories. These results demonstrate that within the first 6 hrs following axonal injury, there were changes in the phosphorylation of proteins associated with pathways involving intracellular signal transduction via MAPK-ERK-1, glutamate/Ca^2+ ^signaling, TNFα activity and transcription factors. The variety of the detected phosphoproteins indicated that signaling pathways from the plasma membrane to the nucleus become activated through phosphorylation events. We further investigated these identified pathways because of their relevance to initiation of cell death and/or regulation of cell survival.

**Table 2:** Selected phosphoproteins identified in the neural retina after axonal injury*.

**Accession Number**	**Protein**	Direction**	**Description**
**Calcium/Potassium channels and regulators**			
IPI00407939	GRIA1	Up	Glutamate receptor, ionotropic, AMPA1
IPI00467841	CALM1	Up	Calmodulin 1
IPI00118978	HCN1	Up	Hcn1 Potassium/sodium hyperpolarization-activated cyclic nucleotide-gated channel 1
IPI00409286	TRPM4	Down	Trpm4 Isoform 1 of Transient receptor potential cation channel subfamily M member 4
IPI00752412	ATP1A3	Down	ATPase, Na+/K+ transporting, alpha 3 polypeptide
IPI00132450	RGS10	Up	Regulator of G-protein signaling 10
IPI00122549	VDAC1	Up	Isoform1 of VDAC Voltage-dependent anion-selective channel 1
			
**Signal Transduction**			
**TNF-alpha pathway**			
IPI00126078	JAK1	Up	Tyrosine-protein kinase JAK1
IPI00122372	TTRAP	Up	TRAF and TNF receptor-associated protein
IPI00124590	CASP8AP2	Down	Casp8ap2 CASP8-associated protein 2
IPI00348883	CARD9	Down	Card9 Novel protein containing a caspase recruitment domain
IPI00108549	SDCCAG3	Down	Sdccag3 serologically defined colon cancer antigen 3 isoform 1
IPI00124414	DAB2IP	Down	Dab2 interacting protein (ASK-interacting protein1)
			
**MAPK-ERK-1 Pathway**			
IPI00755254	OSBP	Up	Similar to Oxysterol-binding protein 1
IPI00111234	MAGI3	Down	Magi3 Membrane-associated guanylate kinase-related MAGI-3
IPI00171977	USP8	Up	Ubiquitin carboxy-terminal hydrolase 8
IPI00170221	ARHGEF6	Up	Rac/CDC42 exhange factor (GEF) 6
			
**Nuclear Activity**			
IPI00753411	SYNE2	Up	Similar to spectrin repeat containing, nuclear envelope 2 isoform a
IPI00460716	JMJD1A	Up	Isoform 1 of JmjC domain-containing histone demethylation protein 2A
IPI00461474	SYNE1	Up	Synaptic nuclear envelope 1, full insert sequence
IPI00228557	TCF20	Up	TCF20 (transcription factor 20) – SPBP
IPI00331063	XPO4	Up	Exportin-4
IPI00751267	H2AO	Up	Similar to H2A histone family, member O
IPI00462466	SETD2	Up	SET domain containing 2-Huntington interacting protein B (Histone methyltransferase)
IPI00761953	SFRS2IP	Up	Splicing factor, serine arginine rich 2 interacting protein
IPI00750315	DCP2	Up	PREDICTED: similar to mRNA decapping enzyme 2
IPI00458583	HNRPU	Up	Heterogeneous nuclear ribonucleoprotein U
IPI00136883	PTBP1	Up	hnRNP I Heterologous nuclear ribonuclear protein I
IPI00421190	STAG2	Up	Stromal Antigen 2
IPI00119301	HES6	Up	Transcription cofactor HES6

*Listing the proteins in Table 2 was based upon the following criteria: 1) the underlying peptides were detected at least twice in the data, 2) the identified protein has known phosphorylation sites in the Phosphosite database [50], and 3) the phosphoprotein levels changed (up/down) by approximately two-fold after axonal injury.

**Direction is the up or down change in phosphopeptide level based upon the average of the 30, 60 min and 6 hrs values divided by the control value.

### Elaboration of signaling pathways detected by phosphorylation screening after optic nerve injury

#### ERK-1 signaling

Although the phosphoproteomics method did not detect phosphorylated ERK-1 (Map kinase 1), we detected the phosphorylation of regulators of ERK-1 activation (Table 2). For example, OSBP (oxysterol binding protein) regulates the activation of ERK-1 by way of binding to phosphatases that act on phosphorylated ERK-1 [11], MAGI-3 regulates activation of ERK-1 by lysophosphatidic acid (LPA) [12] and USP8, also known as UBPY, is a deubiquination enzyme that promotes degradation of the epiderminal growth factor receptor [13], which is a known activator of the ERK-1 pathway.

**Figure 1:**
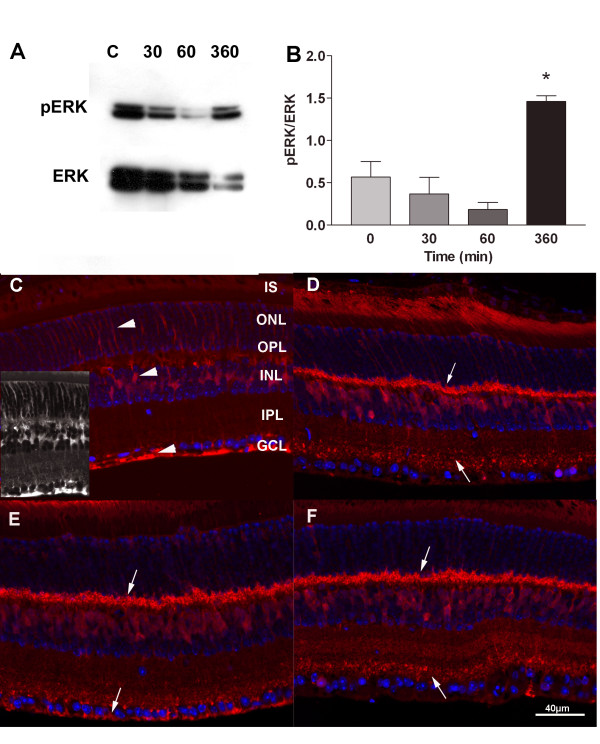
**Activation of ERK-1 (MAPK1)**. A. Western blots were probed with anti-ERK-1 (pThr202/pTyr204) antibody. There was constitutive activation prior to optic nerve crush. However, the phosphorylation ratio (pERK/ERK) increased further at 360 min although the total ERK extracted was lower. B. Graph of the data in A normalized to the total ERK-1 protein. C-F Immunohistochemistry of activated ERK-1, at 0, 30 min, 60 min, and 360 min (6 hr) after optic nerve crush, respectively. Increased labeling of cells in the OPL (down arrows) are particularly evident at 30 min as well as decreased labeling of Muller cells that extend the from the ONL to the GCL (arrowheads). Also note that there is immunolabeling for pERK-1 in the inner stratum of the inner plexiform layer at 30 min and 6 hrs (up arrows) and labeling in the ganglion cell layer at 60 min (arrowheads). The grayscale inset in C shows the staining of the Muller cell marker glutamine synthase. Red = pERK-1, Blue = DAPI.

Therefore, we tested for the activation of ERK-1. By immunoblot, activated ERK-1 (pERK1) appeared to decrease at 30 and 60 min after optic nerve injury, but increased significantly at 6 hrs (Figure 1A–B). Immunohistochemistry showed activated ERK-1 primarily in the Muller cells of control retinas (Figure 1C). However, after optic nerve injury, the distribution of pERK-1 changed dramatically. At 30 min, pERK-1 was no longer detected in Muller cells but now appeared in the OPL and the inner layer of the IPL (Figure 1D). Immunolabeling for pERK-1 in the OPL, which contains photoreceptor synapses, was present at 30 min and persisted for at least 6 hrs after optic nerve crush (Figure 1D–F). The increased labeling in the outer plexiform layer suggests that there were signals to the photoreceptors within 30 min following optic nerve crush. There was also immunolabeling for pERK-1 in the inner stratum of the inner plexiform layer at 30 min and 6 hrs and labeling in the ganglion cell layer at 60 min. This dynamic, cellular redistribution of activated ERK-1 following optic nerve injury is striking and suggests that other cells in the retina are rapidly responding to the axonal injury of the RGCs.

#### Glutamate/calcium signaling

**Figure 2:**
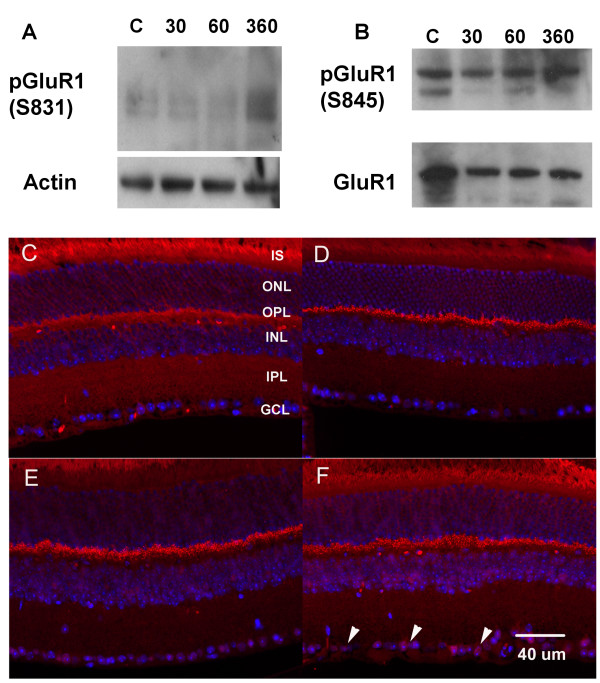
**Phosphorylation of GluR1**. A. Phosphorylation of GluR1 at Ser-831 increased at 6 hrs after optic nerve crush. B. GluR1 protein levels remained relatively constant, while phosphorylation of Ser-845 increased by 6 hrs. C-F. Immunohistochemistry of phosphorylated GluR1 (Ser-845) at 0, 0.5, 1, and 6 hrs after optic nerve crush. Increased pGluR1 is apparent in the ganglion cell layer (arrowheads) at 6 hrs after optic nerve injury. Red, = pGluR1, Blue = DAPI. IS, inner segment; ONL, outer nuclear layer; OPL, outer plexiform layer; INL, inner nuclear layer; IPL, inner plexiform layer; GCL, ganglion cell layer.

After optic nerve crush, we detected phosphorylation of calmodulin (CALM1), the ionotropic glutamate receptor-channel, GluR1 (GRIA), and other ion transport-related proteins (Table 2). These results indicated altered calcium signaling and increased activation of glutamate receptors. Phosphorylation of the GluR1 occurs on tyrosine and serine residues [14]. Using lysates and membrane fractions from whole retinas, we performed immunoblots with site specific phosphoserine antibodies to GluR1. The Ser-831 and Ser-845 sites are adjacent to a putative tyrosine phosphorylation site (Tyr-812) in the carboxyl-terminal domain of the receptor [14]. Increased phosphorylation of Ser-831 was evident at 6 hrs (Figure 2A). Phosphorylation of Ser-845 was detected in the membrane pellet fraction (Figure 2B) and was also increased at 6 hrs. Immunohistochemistry was used to localize where in the retina was phosphorylation of GluR1 occurring following optic nerve crush. As shown in Figure 2C–F, there was increased labeling of GluR1 in the ganglion cell layer by 6 hrs after axonal injury; whereas the labeling in the outer plexiform layer remained relatively constant. The increased phosphorylation of GluR1 by 6 hrs was consistent using proteomics and immunohistochemistry.

#### TNFα signaling

**Figure 3:**
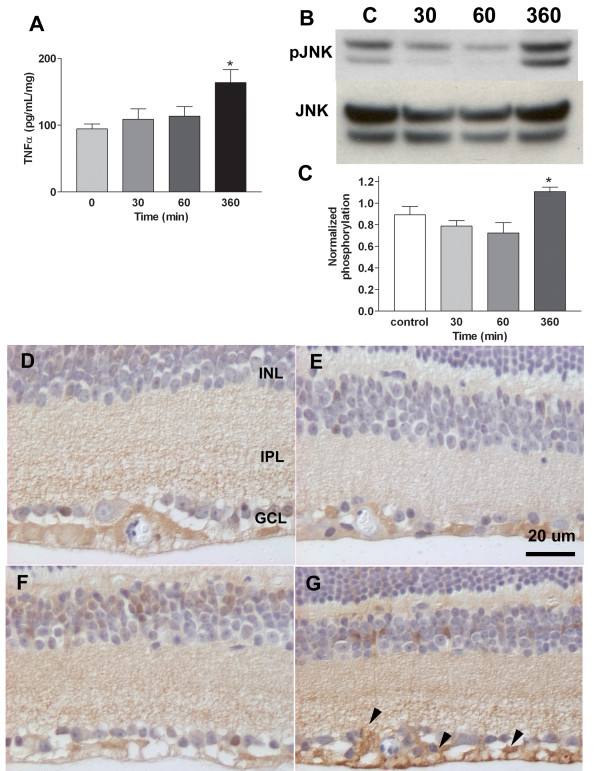
**Activation of TNFα pathways**. A. Detection of TNFα by ELISA in the neural retina. Cytokine levels were determined in three samples at each time point. The level at 6 hr was significantly higher. B-C. Detection of phosphorylated JNK in the neural retina. B. Western blots (upper image) were probed with an anti pJNK antibody. C. ELISA confirmed the change in activation of JNK at 6 hrs after optic nerve injury. Data are normalized to total protein. D-G. Immunohistochemistry of activated JNK in the neural retina. Antibodies to pJNK were used on sections at 0, 30 min, 60 min and 6 hr after optic nerve crush. Increased labeling is evident in the GCL (arrows) at 6 hr.

In our phosphoprotein screen, several proteins related to the TNFα pathway were detected following optic nerve crush (Table 2). Therefore, we determined in the retina the presence of the ligand and downstream protein kinases that can be activated through the TNFα pathway. As shown in Figure 3A, TNFα was detected in control retinas but TNFα levels increased markedly by 6 hrs after optic nerve injury.

If the TNFα pathway was activated, two major intracellular signaling pathways may also be activated: SAPK/JNK and NFκB. The activation of SAPK/JNK over the 6 hrs time course is shown in Figure 3B–C. Consistent with the increased levels of TNFα by 6 hrs, there was a significant increase in activated SAPK/JNK by 6 hrs after optic nerve injury. By immunohistochemistry, pJNK was found throughout the inner retina under control conditions and at 30 and 60 min post optic nerve crush (Figure 3D, E and 3F). However, by 6 hrs after optic nerve injury (Figure 3G), increased activation of JNK was present within the ganglion cell layer, which includes the RGCs. Thus, the proteomics, immunoblots, ELISA and immunohistochemistry all identify activation of the TNFα pathway, most likely in the RGCs, by 6 hrs following optic nerve injury.

We assayed for the activation of NFκB by means of a specific ELISA for phospho-Ser32 on the IκB subunit of NFκB, immunoblot for phosphorylated protein and phosphorylation of the p65 subunit of NFκB. None of these assays demonstrated activation of the NFκB pathway by 6 hrs following optic nerve crush (data not shown). Other phosphoproteins related to TNFα signaling (Table 2) may be responsible for the down-regulation of NFκB (See Discussion).

#### Nuclear activity

Active transcription involves the dynamic, post-translational modification of histones and other proteins associated with chromatin, as well as transcription factors that translocate to the nucleus depending upon their phosphorylation state. Table 2 contains several transcriptional cofactors, such as TCP20, and HES6. TCP20 (also known as SPBP- streomelysin-1 platelet-derive growth factor responsive element binding protein) enhances the activity of various transcription factors, including c-Jun [15]. HES6 is a basic helix-loop-helix transcription factor that promotes neuronal differentiation [16] but inhibits astrogenic differentiation [17]. HES6 is phosphorylated by ERK-1 which is necessary for its anti-astrogenic activity [17].

**Figure 4:**
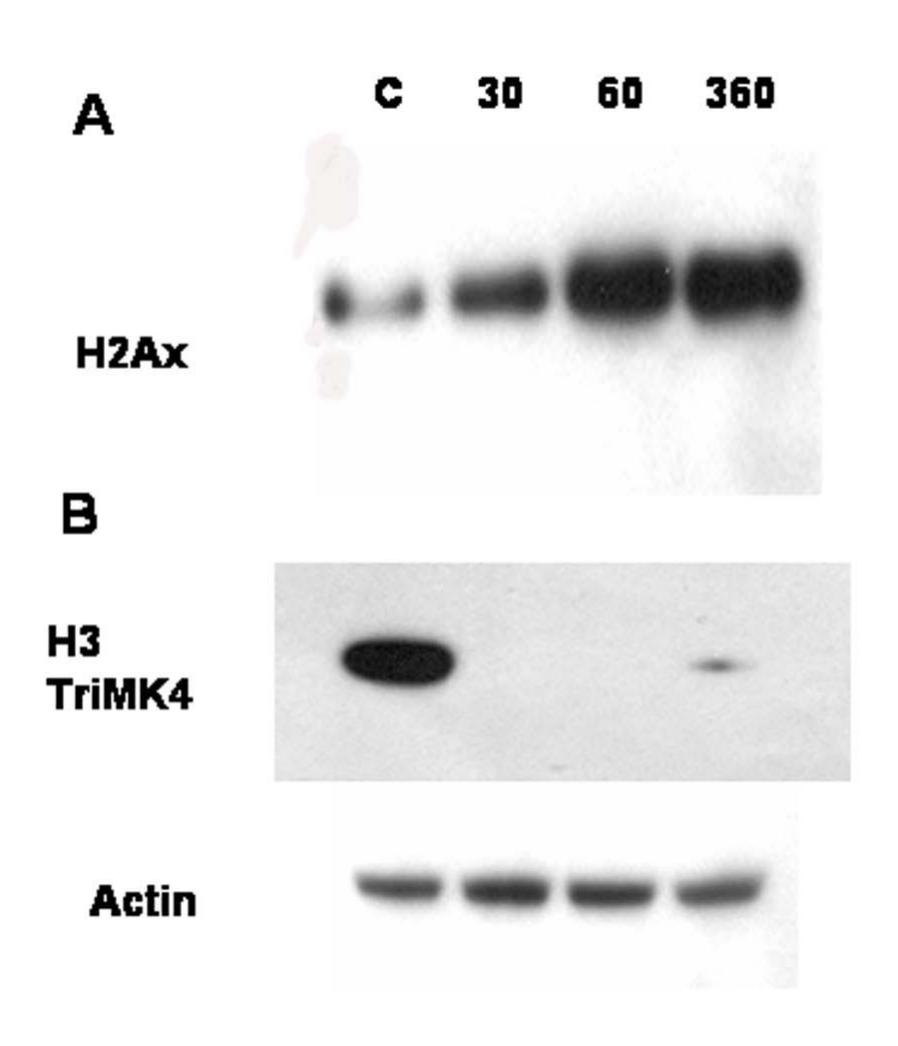
**Histone phosphorylation and methylation**. Western blots probed with A. antiphospho-H2A (Ser-139) and B. trimethyl-K4 H3 histone. Phosphorylation of H2A increased while methylation of histone H3 (at lysine-4) decreased after optic nerve injury. The demethylation of H3 persisted for several hours and started to recover at 6 hr post injury. Actin served as a loading control in these experiments.

In our survey for nuclear phosphoproteins based on mass spectrometry, we also found that H2A, JMJD1A (a histone demethylase) and SETD2 (a histone methylase) were phosphorylated within 6 hrs after optic nerve injury. Using an antibody to phosphorylated H2A (pSer139), we confirmed the phosphorylation state of this histone in the neural retina following optic nerve injury. Western blotting indicated that H2A is phosphorylated and that the level of phosphorylation increased during the 6 hrs after optic nerve injury (Figure 4A). The phosphorylation of H2A may be related to chromatin remodeling [18,19] and is also a feature of the initiation of apoptosis [20,21].

Because we detected phosphorylation of two proteins involved in histone methlation/demethylation (JMJD1A and SETD2 in Table 2), we further investigated selected histone H3 methylation sites. To detect changes in histone methylation, we used an antibody directed towards histone H3 di and tri-methylated at K4. Trimethylation at this site is associated with the active transcription of a number of genes [22]. As shown in Figure 4B, H3-K4 trimethylation is constitutive, but decreases abruptly by 30 min, and recovers slightly at 6 hrs after optic nerve crush. H3-K4 dimethylation also decreased with time after injury (data not shown). Thus, the increase in H2A phosphorylation and changes in histone H3 methylation may be linked to decreased transcription of certain genes within 6 hrs following optic nerve crush.

### Differential gene expression in the ganglion cell layer post retinal injury

To examine the early changes in gene expression within the ganglion cell layer after optic nerve crush, we performed laser capture microdissection on retinal tissue sections to extract material from the ganglion cell layer. This layer contains RGCs as well as astrocytes, displaced amacrine cells, microglia, vascular endothelia and the processes from Muller cells. Thus, laser capture microscopy enriched our sample for RGCs compared to having used full thickness retina. mRNA was prepared from ganglion cell layer samples obtained from retina sections of eyes with optic nerve crush and compared to ganglion cell layer samples from control retina sections. Samples were subjected to linear amplification and processing, followed by hybridization to a mouse microarray (Affymetrix Mu430 V2). A list of 220 differentially expressed genes was obtained after filtering for a minimum 1.2- fold change and p < 0.05 (Additional file 2). Changes in selected genes were verified using qRT-PCR. We used gene ontology analysis (GoMiner and ProfCom) to analyze changes in gene expression with regard to cellular signaling.

In the gene ontology analysis, expression of genes involved in transcription and transcriptional regulation were one of the most significant with 31 differentially expressed genes (p = 0.01, Additional file 3). The increase in nuclear gene phosphorylation that we found with phosphoproteomics apparently leads to alterations in transcriptional activity. Another category containing significantly altered gene expression was phosphorylation with 16 differentially expressed genes (p = 0.0035, Additional file 3). Table 3 contains a curated list of genes we found in these GO categories grouped with respect to functions in signal transduction, Ca^2+ ^homeostasis and cell death.

**Table 3:** Differential expression of selected genes in the ganglion cell layer 6 hrs after optic nerve injury

			Genbank	Fold	
Group	Gene Symbol	Description	Accession	Change	p-value
**Signal transduction**					
	Socs3	Suppressor of cytokine signaling 3	BB241535	3.33	0.0018
	Gsk3b	Glycogen synthase kinase 3 beta	BG063622	1.39	0.0454
	Akap9	A kinase (PRKA) anchor protein 9	BB109183	1.59	0.0165
	Pdgfra	Platelet derived growth factor receptor -alpha	AW537708	1.50	0.0325
	IL20rb	Interleukin 20 receptor beta	BE852308	1.84	0.0371
	Prkcbp1	Protein Kinase C binding protein 1	AK014397	1.36	0.0417
	Mast2	Microtubule associated serine/threonine kinase 2	NM_008641	1.30	0.0319
	Map3k10	Mitogen activated protein kinase kinase kinase 10	AI851771	-1.48	0.0363
					
**Calcium tansport/homeostasis**					
	Camk2a	Calcium/calmodulin-dependent protein kinase II alpha	AW490258	1.32	0.0245
	Cacng2	Calcium channel, voltage-dependent, gamma subunit 2	BB342913	1.26	0.0298
	Itpr2	Inositol 1,4,5-triphosphate receptor 5	BC013815	-1.44	0.0102
	Trpc2	Transient receptor potential cation channel, subfamily C, member 2	BC003841	1.24	0.0318
	Gcm2	Glial cells missing homolog 2	AF081556	1.37	0.0224
					
**Cell Death**					
	Bax	Bcl2-associated × protein	BC018228	1.32	0.0275
	Faim3	Fas apoptotic inhibitory molecule 3	AK007714	-2.48	0.0118
	Atf2	Activating transcription factor 2	BM119623	1.25	0.0489
	Ercc1	Excision repair cross-complementing rodent repair deficiency1	AW538163	-1.27	0.0155
	Foxp1	Forkhead box P1	BM220880	1.27	0.0285
	Nfatc4	Nuclear factor of activated T-cells, calcineurin-dependent 4	AV166177	-1.65	0.0387
	Rasa1	RAS p21 protein activator	AA124924	1.23	0.0390
	Rtn4	Reticulon 4	BE988775	1.31	0.0187
	Ski	v-Ski sarcoma viral oncogene homolog	AV381512	1.33	0.0371
	Smad5	SMAD, mothers against DPP homolog 5	U77638	-1.7	0.0250
	Aifm3	Apoptosis inducing factor, mitochondrion-associated 3	BF320633	1.31	0.0221

We also found that expression of genes related to cytokine/growth factor signaling was altered at 6 hrs post injury. Socs3 and Atf2 were upregulated and Map3K10 was downregulated (Table 3). Atf2 is a transcription activator downstream of JNK, while Map3K10 is an activating kinase upstream of JNK. Consistent with the microarray results, qRT-PCR data confirmed the upregulation of Socs3 (4.4-fold). Several differentially expressed genes at 6 hrs post injury were associated with regulation of cell death pathways: Aifm3 and Bax are directly associated with mitochrondria permeability channels that, when opened, leads to apoptosis. We confirmed the upregulation of Bax expression (1.6-fold) using qRT-PCR.

**Figure 5:**
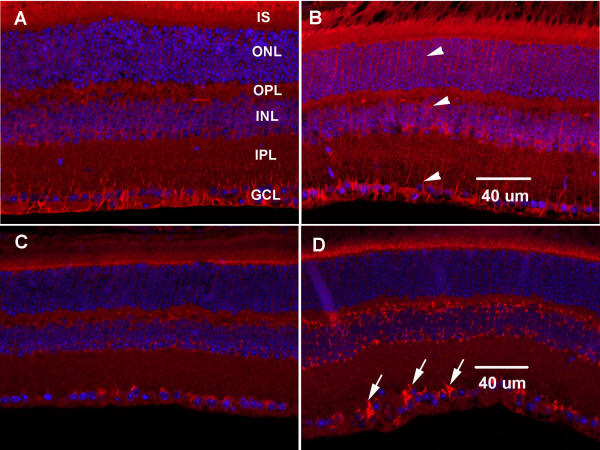
**Immunohistochemistry of differentially expressed proteins**. A-B: Expression of SOCS3 and C-D: BAX in the control and 6 hrs after optic nerve crush. SOCS3 was expressed throughout the retina and appeared increased in Muller cells (arrowheads) after optic nerve injury. Increased BAX labeling after optic nerve injury was particularly evident in the ganglion cell layer (arrows), consistent with pending programmed cell death of RGCs. Red = target antibody, Blue = DAPI.

Using immunohistochemistry, we investigated which cell types are differentially expressing SOCS3 and BAX proteins at 6 hrs after optic nerve injury. As seen in Fig. 5A–B, SOCS3 appears to be present throughout the retina but is increased at 6 hrs, particularly in the Muller cells. Similarly, BAX increased at 6 hrs, particularly in the ganglion cell layer (Fig. 5C–D). Thus, the immunohistochemistry demonstrating proteins was consistent with the mRNA expression data. Expression of other cell death genes was also apparent at 6 hrs (Table 3). Consistent with the lack of activation of the NFkB pathway, we did not observe upregulation of the anti-apoptotic factor Bcl-2 or caspase inhibitors [23]. Thus, changes associated with the initiation of programmed cell death may already have started in RGCs within 6 hrs after optic nerve injury.

## Discussion

We used a multidisciplinary approach to investigate the temporal, intercellular and intracellular signaling that immediately follow optic nerve injury. Our hypothesis was that there are cellular events in different cells in the retina very early after optic nerve injury. Our intent was not to investigate all pathways or any one pathway in-depth, but to find multiple signaling pathways, that would be representative of sequential changes. Our data provide a temporal, sequential framework of early events within the first 6 hrs after optic nerve injury. Previous studies have investigated changes in selected protein phosphorylation [1] or gene expression [2,3] at one day to several weeks after optic nerve injury. Thus, our data provides the first observation of responses in the neural retina as early as 30 min after axonal injury.

As answers to the questions that were raised in the Introduction of this paper, we believe that:

1. The soma of the RGC "senses" that its axon has been injured within 30 min. This interpretation of our data is based on the dramatic de-activation of the phosphorylation state of ERK1 in the Muller cells within 30 min. Muller cells and astrocytes express activated ERK-1 in the retina, and these cells express higher levels in retinas from glaucomatous donors [24]. Muller cells wrap around the somas of RGCs and have many points of contact. We hypothesize that within 30 min, the somas of the RGCs signal the Muller cells, which lead to loss of the activation/phosphorylation state of ERK1 in the Muller cells. If there are changes in Muller cell activity by 30 min post axonal injury, the likely source of the signals to affect these changes is the somas of the RGCs with damaged axons. Thus, the somas of the RGCs have presumably "sensed" that a cellular event has occurred.

2. The somas of the RGCs probably signal that a catastrophic event has occurred to many neurons and glia in the retina within 30 min. Surprisingly, 30 min after acute injury, when Muller cells have lost ERK-1 activation/phosphorylation, ERK-1 activation/phosphorylation simultaneously appears in the outer plexiform layer, the location of the photoreceptor synapses, in the inner nuclear layer and in the inner plexiform layer.

3. Cell death signals are apparent within 6 hrs following injury to the RGC axon. These death signals include: an increase in TNFα production and changes in phosphorylation of related TNFα pathway proteins (CASP8AP2 (Flash), TTRAP, SDCCAG3, JNK, CARD9, and DAP2IP). DABIP2 (AIP1) [25,26] is directly involved in signaling from the TNFα receptors to activate JNK, while SDCCAG3 is involved in receptor trafficking [27]. These signals may not be sufficient to induce cell death at 6 hrs but may be part of the early events that lead to programmed cell death. The lack of activation of the NFκB survival pathway is consistent with changes in the phosphorylation of other TNFα pathway components, (eg TTRAP, CARD9, CASP8AP2) that can negatively regulate NFκB activation [28-30]. Finally, protein kinase MAST2, which is upregulated 6 hrs post optic nerve crush (Table 3), interacts with TRAF6 in the TNFα pathway so as to decrease activation of NFκB [31].

4. There are nuclear events causing new protein synthesis within 6 hrs following injury to the RGC axon. Two new proteins, for example, BAX and AIFM3, are associated with the initation of programmed cell death; whereas, another, RTN4, sequesters the antiapoptotic BCL-2 protein [32]. The decrease in histone methylation (Figure 5) and the upregulation of transcription factors (Table 3) at 6 hrs after optic nerve crush are consistent with changes in transcriptional activity. Furthermore, phosphorylation of H2A at Ser-139 is associated with release of H2A from the nucleus and apoptosis [18].

The increase in expression of Socs3 can be related to JAK1 activation [33] and cytokine induced degeneration. For example, Socs3 is also upregulated in the neural retina upon light-induced injury [34]. In these studies, the activity of one or more of the IL-6 family of cytokines (IL-6, CNTF, NP, LIF, CLC) was the stimulus for Socs3 upregulation. We did not detect upregulation of any single member of the IL-6 family in our microarray data. Thus, as with light-induced injury, multiple cytokines likely lead to the increase of Socs3 expression.

The increased expression of Bax and other genes associated with programmed cell death is consistent with the beginnings of a pro-apoptotic program that eventually translocates BAX [5,6] and the related proapoptotic protein BIM [4,35] to the mitochondria to induce death in RGCs. Upregulation of BAX protein has been shown to persist after optic nerve crush [1,6]. Although a detailed promoter analysis has not been reported, BAX upregulation has been linked to JNK activation [36] that we observed within 6 hrs of optic nerve injury. Bax knockout mice are more resistant to RGC cell death after optic nerve crush, but not to degeneration induced by glutamate excitotoxicity [37]. RGC cells in Bim knockout mice are also protected from optic nerve axotomy-induced death [4]. The proapoptotic activity of Bim is negatively regulated by ERK-1 phosphorylation, while phosphorylation by JNK enhances Bim activity possibly by dissociation from intracellular sequestration [38]. Phosphorylation of BIM by ERK-1 causes its degradation by the proteosome [39] so that the regional differences we observe in ERK-1 and JNK activation (Figs 3 and 4) could affect Bim levels in various cell types.

In addition, we note that there is limited survival signaling in the retina immediately after optic nerve injury. Previous studies have shown that survival signaling by IGF-1 through the phosphoinositide-Akt pathway begins to decrease within two days after optic nerve crush [1]. The loss of IGF-1 signaling may be due to the upregulation of Socs3 (Table 3) which is known to antagonize this pathway [40] and interacts directly with the IGF-1 receptor [41]. The changes in glutamate receptor phosphorylation that we observed after optic nerve crush suggests that altered Ca^2+ ^signaling is part of the degenerative process. Brain-derived neurotrophic factor (BDNF) is an important trophic factor for RGC cells and has been shown to be neuroprotective in RGC injury paradigms (Reviewed in [42]). However, the upregulation of Camk2 and related Ca^2+ ^signaling (Tables 2 and 3) antagonizes the trophic activity of BDNF [43]. Thus, application of BDNF, IGF-1, and related factors [44] may be of only short-term benefit after optic nerve injury.

Based on our data we offer the following hypothesis: The soma of the RGC "senses" that its axon is damaged within 30 min and signals the Muller cells, which, we believe, signal the entire retina that a catastrophic event has occurred. Furthermore, within 6 hrs of damage to the optic nerve, death signals are present in the retina that will ultimately lead to RGC degeneration. The temporal rapidity with which these events occur suggest that attempting to interfere with programmed cell death at a later time may be fruitless and, perhaps, not possible

### Experimental Procedures

#### Animal model

Retinas were obtained from male C57BL/6J mice. All experiments were performed in accordance with the U.S. Public Health Service Policy on Humane Care and Use of Laboratory Animals, the National Institutes of Health Guide for the Care and Use of Laboratory Animals, the ARVO Statement for the Use of Animals in Ophthalmic and Vision Research, and institutional, federal, and state guidelines regarding animal research. Groups of 5–9 mice were subjected to optic nerve crush. Axons of the optic nerve were crushed with fine forceps for 10 sec, 2 mm posterior to the globe, under direct visualization, within an intact meningeal sheath. By performing the optic nerve crush 2 mm posterior to the globe, the injury is distal to the entry of the ophthalmic artery into the optic nerve. Thus, care is taken to not disturb the retinal blood supply. Optic nerve crush has been widely used by many laboratories and is well documented in the literature [45,46]. At the desired times (30, 60 and 6 hrs after crush) eyes were enucleated and neural retina removed and frozen at -80°C. Controls (0 min) were contralateral eyes that had not been injured from the same animals in each group.

#### Preparation of retinal extracts

Retinal tissue (55–100 mg) was homogenized in 150 μl of lysis buffer (20 mM Tris-HCl, pH 7.4, containing 2 mM EDTA, 150 mM NaCl, 1% (v/v) NP-40, multiple protease inhibitor cocktail (Roche Applied Science, Indianapolis, IN) and 1 mM sodium orthovanadate). A motorized pestle was used in 3 × 20 sec bursts on ice. The mixture was centrifuged at 15,800 × g for 20 min at 4°C. The supernatant was removed and the pellet re-extracted with 50 μl of lysis buffer with mixing by up and down action of a pipette. The mixture was centrifuged again and the supernatants combined. Protein concentrations were measured (BCA reagent, Pierce Chemical Co.).

### Strategy

The initial screening for changes in phosphorylated proteins was done using affinity capture methods coupled to mass spectrometry for protein identification. These methods included anti-phosphotyrosine beads for enrichment of tyrosine phosphorylated proteins followed by separation of the captured material using one-dimensional gel electrophoresis. We used metal-ion chelate chromatography of phosphopeptides obtained from proteins that were not captured by anti-phospho-tyrosine antibodies. We did these experiments at multiple points post injury to attempt to capture a broad spectrum of events in cellular signaling. Although the method used was only semi-quantitative, it lends itself to detection of changes in multiple phosphoproteins for each experimental time point.

#### Isolation of phosphoproteins/peptides

Tyrosine-phosphorylated proteins were isolated by immunocapture with anti-phosphotyrosine antibody 4G10 conjugated to agarose beads (Millipore Billerica, MA). This antibody has been used previously to characterize tyrosine phosphoryated proteins in stimulated cell systems [47] and for quantitative phosphoprotein detection [48]. Lysate containing 1 mg of protein was diluted 1:10 with lysis buffer without NP-40 detergent and applied to 50 μl of a slurry of 4G10-conjugate. The antibody binding reaction was incubated at 4°C with gentle rocking for 16 to 24 hr. Beads were pelleted by centrifugation (2000 rpm, 3 min). The supernatant was removed and saved for subsequent digestion and isolation of additional phosphopeptides (see below). The beads were washed two times with 50 μl of lysis buffer without NP40 and the washings combined with the original supernatant. The beads were washed with lysis buffer (500 μl) without NP-40 and the supernatants discarded. Proteins were eluted from the beads by applying 50 μl of SDS-PAGE sample buffer (Invitrogen) and heating to 95°C for 10 min. After brief centrifugation, the supernatants were removed and applied to individual lanes of a 4–12% polyacrylamide gel (Biorad Criterion, Hercules, CA) and electrophoresed at constant voltage. Gels were stained with Simply Blue stain (Invitrogen, Carlsbad, CA) and de-stained in water.

### In gel digestion of phosphotyrosine antibody-captured proteins

Each gel lane was cut into 10 bands (4 mm × 6 mm) and further chopped into ~1 mm pieces and transferred to 1.5 ml Eppendorf tubes. Gel pieces were washed with 50 mM ammonium bicarbonate, 50% acetonitrile solution (3 × 15 min), and then in 100% acetonitrile. After removal of the solvent and drying in a Speed Vac (Thermo-Fisher, Pittsburgh, PA) concentrator, gels were rehydrated with 70–80 μl of 50 mM ammonium bicarbonate containing 0.01% (w/v) trypsin (Promega, Madison, WI). After incubation at 37°C (16–24 hr), the reactions were stopped by adding 1 volume of 5% (v/v) trifluoroacetic acid. The supernatants were removed and gel pieces further extracted twice with 100 μl of 0.1% trifluoroacetic acid/60% acetonitrile for 30 min. Combined extracts were then evaporated to dryness with a Speed Vac concentrator. The residues were dissolved in 20 μl of 0.1% formic acid/10% v/v acetonitrile).

#### Isolation of additional phosphopeptides from retinal extracts

The flow-through or non-bound fraction from the antiphosphotyrosine capture step (above) was denatured by addition of an equal volume of 6 M guanidine hydrochloride solution. Protein disulfides were reduced with triscarboxyl-ethylphosphine (Sigma-Aldrich, St. Louis, MO) (12.5 mM) at room temperature for 1 hr. To each sample, iodoacetamide (in acetonitrile) was added to a final concentration of 25 mM and the reactions incubated in the dark for 1 hr. The solution was then transferred to a dialysis cassette (10,000 MWCO) and dialyzed against 50 mM ammonium bicarbonate (1 L) at 4°C. The dialysis buffer was changed 3–4 times over 24 hr. The retained fraction was then concentrated in the Speed Vac to 0.5 ml and then trypsin was added to a final concentration of 0.01% and incubated at 37°C for 20 hr. The reactions were stopped by adding 10 μl of acetic acid. The reactions were dried on a Speed Vac concentrator and re-dissolved in 200 μl of 0.1% formic acid, 10% acetonitrile. The OD280 of each solution was measured after 100-fold dilution with water. A volume equivalent to 150 OD280 units of each sample was then diluted to 200 μl with 5% acetic acid and applied to a Ga-conjugated phosphopeptide isolation cartridge (Pierce, Rockford, IL) that had been rehydrated as per the manufacturer's instructions. The flow-through from the cartridge was re-applied and then washed with 200 μl of 0.1% acetic acid, 2 × 100 μl of 0.1% formic acid/10% acetonitrile. Peptides were eluted with 2 × 75 μl applications of 0.1 M ammonia in 10% methanol. One hundred μl of 5% acetic acid was then added to the eluates and the samples evaporated to dryness on the Speed Vac concentrator. The residue was re-dissolved in 25 μl of 0.1% formic acid/10% acetonitrile.

### Mass Spectrometry

Peptides from the gel bands of tyrosine phosphorylated proteins were separated on a nano-flow column (0.075 × 50 mm, C18 Agilent Santa Clara, CA). The column was eluted at 0.3 μl/min with a gradient of 20 to 65% (v/v) acetonitrile in 0.1% formic acid. Peptides from the phosphopeptide isolation cartridge were separated using automated two-dimensional chromatography on the Agilent 1100 chromatography system. In the first dimension, phosphopeptides were injected onto a sulfated-ion exchange column (PolyLC 0.3 mm × 50 mm (Columbia, MD). Peptides were then eluted with steps of 0.05, 0.1, 0.2 and 0.4 M ammonium acetate onto a nanoflow column (0.075 mm × 150 mm, Agilent). The column was eluted at 0.3 μl/min with a gradient of 5–65% acetonitrile in 0.1% formic acid. Peptides were detected using an Agilent 1100 XCT ion-trap LC-MS system as described previously [49]. Each sample from the gel band isolation was run twice.

### Data Analysis

Peptide mass and fragmentation data were filtered and database searching done using Spectrum Mill software (Agilent) as described previously [49]. We used the mouse International Protein Index (IPI) database for searching the mass spectrometry data generated from each sample. From each sample run, a curated list of hits was obtained. This list was based upon the database score and the quality of mass spectral data. For comparisons, we used only those hits that were identified at least twice in each sample. The MS-data intensity values for each peptide were then averaged over the three time points (3 0 min, 60 min, 6 hrs) and divided by averaged control values. The final list of identified proteins is summarized in Additional file 1. This spreadsheet contains the sequences of phosphopeptides identified by mass spectrometry data. Also included is the analysis for the presence of phosporylation sites using Phosphosite [50].

### Immunohistochemistry

Enucleated eyes were fixed in 2% wt/vol paraformaldehyde in 0.01 M phosphate buffered saline (PBS; pH 7.4) at 4°C overnight. Six animals were used for each group in all immunohistochemistry experiments. Immunohistochemistry was performed on paraffin sagittal sections of retina for pJNK (Cell signaling, Danvers, MA) using the Vectastain Elite ABC kit (Vector Laboratories, Burlingame, CA) and diaminobenzidine as a substrate. As a negative control, sections were treated in the same manner, except that incubation with primary antibody was omitted. The sections were treated for equal time in DAB reagent and photographed at the same time. Phosphorylated GluR1 (Ser-845, Zymed, Billerica, MA), phosphorylated ERK-1 (Cell Signaling) SOCS3 (Santa Cruz P-19, Santa Cruz, CA), BAX (Santa Cruz H103), and glutamine synthase (Chemicon-Millipore) were detected using Rhodamine-labeled secondary antibody (Vector Labs). Nuclei were stained with DAPI. Sections were washed, mounted and viewed under a fluorescence microscope (Olympus, AX70). As a negative control, sections were treated in the same manner, except that incubation with primary antibody was omitted.

### Isolation of retinal ganglion cell layer by laser capture microscopy (LCM)

The LCM system that we used (Veritas Microdissection System, Molecular Devices, Sunnyvale, CA) obviated the need for tissue dehydration prior to microdissection. This enabled us to isolate quiescent retinal ganglion cells directly from 8-μM frozen sections of mouse eyes, thereby increasing the yield and quality of RNA (see below). Frozen sections, mounted on special membrane-coated slides (P.A.L.M. Microbeam), which facilitated the capture of cells, were briefly (1 min) stained with hematoxylin (HistoGene™; Arcturus, Mountain View, CA). In addition to visualizing the retinal structures, this staining procedure also removed the OCT mounting medium. Labeled sections were tracked with intergral light microscope using a 20× objective. Retinal ganglion cells to be isolated were outlined with a "light" pen or cursor on a monitor screen. Such an outline defined the area that would be cut and catapulted intact into a Capsure Macro LCM cap (Molecular Devices, Sunnyvale, CA). In this manner, approximately 6000 cells from the ganglion cell layer were isolated from each eye.

### Total RNA Isolation and cRNA Amplification

Total cellular RNA from LCM-captured cells was isolated and purified (Pico-Pure™; Arcturus). Samples of the total starting RNA were analyzed by capillary electrophoresis (Agilent Technologies, Palo Alto, CA) to assess the degree of purification. Approximately 60 ng of total cellular RNA could be extracted from 6000 cells from the ganglion cell layer that were isolated by LCM. When this RNA was contrasted with commercially prepared total RNA from mouse liver using picogram chips and a Bioanalyzer (Agilent Technologies), sharp bands corresponding to the 18 S and 28 S RNA were observed for all samples RNA quality was further assessed by calculating the RNA integrity number, which is based on a proprietary Agilent Technologies algorithm. Total RNA from the isolated cells was subjected to cRNA amplification. Briefly, 1.5 rounds of cRNA amplification were accomplished using a Ribo-Amp^® ^OA RNA amplification protocol (Arcturus). First strand cDNA was generated by reverse transcription using the total RNA. After the second strand cDNA was synthesized, a T7 RNA polymerase-driven cRNA synthesis was performed to obtain the first round of cRNA amplification. A second double strand cDNA synthesis was performed followed by a second round of cRNA amplification. A *BioArray*™ *HighYield*™ RNA transcript labeling protocol (T7) (Enzo Life Sciences, Famingdale, NY) was employed for the second round of amplification to biotinylate the cRNAs. To examine the reaction quality, an aliquot from the first stand cDNA synthesis in the first round cRNA amplification and another from the second strand cDNA synthesis in the second round amplification sample were removed for real-time PCR analysis.

### Microarray Analysis

Amplified RNA (8 μg) was hybridized to Affymetrix (Santa Clara, CA) Mu430 v2.0 chips and processed as recommended by the manufacturer. Three chips each were used for the control and 6 hrs crush samples. For each dataset, invariant set normalization was performed using the PM/MM model for calculating signal intensities in dChip 2006 [51]. Thresholds for selecting significant genes were set at a relative fold-difference of > 1.2, absolute intensity difference between sample and baseline > 80, and paired t-test value of p <0.05. Genes meeting all of these criteria were considered as significantly different. This resulted in a list of 239 differentially expressed genes (160 upregulated and 79 down regulated, Additional file 2). Microarray data have been deposited in the GEO database with the series accession number GSE11862.

### ELISA

ELISA assays for phosphoproteins were done with sandwich ELISA kits (Cell Signaling) following the manufacturer's instructions. These were used to detect phosphoJNK (Thr183, Tyr185) and phospho IκB (Ser32) in soluble tissue extracts. Briefly, extracts were diluted with the assay buffer to the desired total protein concentration (0.25, 0.5, 1.0 mg/ml) to 100 μl and applied in duplicate to the wells of the ELISA plate containing the capture antibody. Controls without added lysate were included in all assays. The plates were covered and incubated at 4°C for 12–16 hrs to allow binding of the target protein to the plate. Wells were then washed 4 times with the wash buffer supplied in the kit. The wells were then covered and incubated with the antiphosphoprotein antibody and incubated for 1 hr at 37°C. The plates were washed again and then incubated with horse radish peroxidase (HRP) linked detector antibody for 30 min at 37°C. The plate was washed again and then incubated with HRP substrate solution for 30 min at room temperature. Stop solution was added and the absorbance of converted substrate read at 450 nm in a plate reader.

Mouse TNFα was measured in soluble tissue extracts using an ELISA kit (Pierce). Briefly, tissue extracts were diluted to 0.25 or 0.5 mg/mL and 50 μl applied in duplicate to the ELISA plate. TNFα standards over the range of 35 to1225 pg/ml were measured in duplicate along with the samples. Then 50 μl of biotinylated antiTNFα antibody was added to all of the wells and the plate covered and incubated for 2 hr at room temperature. The plate was washed 5 times and then the wells were incubated with 100 μl of HRP-streptavidin solution for 30 min at room temperature. The reaction was stopped by adding an equal volume of acidic (2N HCl) stop solution. The absorbance was then read at 450 nm in a plate reader.

## Abbreviations

RGC: retinal ganglion cell; HRP: horseradish peroxidase; IS: inner segment; ONL: outer nuclear layer; OPL: outer plexiform layer; INL: inner nuclear layer; IPL: inner plexiform layer; GCL: ganglion cell layer; NP-40: Nonidet P40 detergent; MS: mass spectrometry; LC-MS: liquid chromatography – mass spectrometry; LCM: laser capture microscopy; RT-PCR: quantitative reverse transcriptase PCR.

## Competing interests

The authors declare that they have no competing interests

## Authors' contributions

ALW performed the axon injury on the control and test animals. YM performed tissue extractions from animal experiments and protein biochemistry. TJL performed mass spectrometry, data analysis and wrote drafts of the manuscript. TJL, ALW and AHN interpreted the collected data. AHN designed the study and edited and approved the final manuscript.

## Supplementary Material

Additional File 1 **Table S1.** S1 is a spreadsheet of all of the identified phosphproteins and phosphopepitde sequences. Click here for file

Additional File 2 **Table S2.** S2 is a spreadsheet with all of the differentially expressed genes (1.2 fold changed p < 0.05) at 6 hrs post axon injury compared to control. Click here for file

Additional File 3 **Table S3. **S3 is a spreadsheet containing the gene. Ontology analysis of the 6 hrs post axon injury differentially expressed genes from S2. Click here for file
